# Catalytic Impedance Spectroscopy: Concept and Application on CO_2_ Methanation

**DOI:** 10.1021/acs.jpclett.4c02442

**Published:** 2024-10-11

**Authors:** Andreas Borgschulte, Marco Achermann, Marin Nikolic

**Affiliations:** † Empa—Swiss Federal Laboratories for Materials Science and Technology, Laboratory Chemical Energy Carriers and Vehicle Systems Laboratory, Überlandstrasse 129, CH 8600 Dübendorf, Switzerland; ‡ Department of Chemistry, Zurich University, Winterthurerstrasse 190, CH 8057 Zürich, Switzerland; § Kantonsschule Reussbühl Luzern, Ruopigenstrasse 40, CH 6015 Luzern, Switzerland

## Abstract

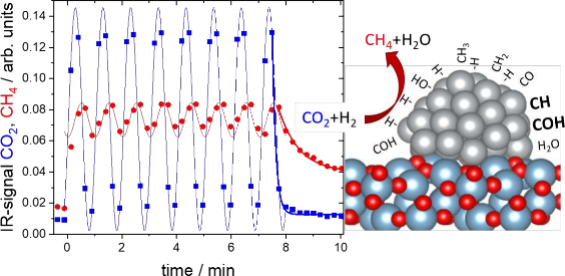

Complex modeling of periodic excitation combined with time-resolved product detection is used to describe the complex response function of a catalytic system. We describe this concept of catalytic impedance spectroscopy (CIS) and the underlying general experimental approach and a concrete setup. The feasibility of CIS is experimentally demonstrated along the catalytic CO_2_ methanation reaction. The measurements confirm the theoretically anticipated rate-determining step of HCO* → CH* on Ni catalysts. Limitations and prospects of CIS to unravel reaction mechanisms are discussed.

The analysis of reaction mechanisms is a pivotal task in catalysis research.^1^ It is usually performed along steady state experiments in which the dependence of the product yield on external parameters such as pressure and temperature is analyzed. Recently, so-called modulation excitation experiments were designed to study the dynamic behavior of intermediates.^2,3^ In this Letter, we extend this idea to develop an analogy of electrochemical impedance spectroscopy for catalysis (catalytic impedance spectroscopy, CIS).

The concept is based on the striking mathematical similarity between electrical currents and chemical flows: the flux *j*_*n*_ of particles of species *n* driven by a chemical reaction and the flux *j*_*c*_ of charged particles such as electrons and ions *c* driven by an electric potential can be described by the same math (Onsager theorem):^4^
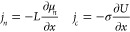
1where μ_*n*_ and *U* are the chemical and electric potentials of particles and charged particles such as electrons and ions, respectively. σ is the conductivity, and *L* represents the transport coefficient(s). The second equation represents Ohm’s law. A simple chemical reaction without enthalpy change is diffusion; defining *L* = *D*/(*RT*)·*c*_*n*_ results in Fick’s law
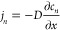
and gives *L* a physical meaning (*c*_*n*_ is the local concentration). The equations can be combined, which is useful in electrochemistry as the chemical potential of a charged particle is nothing else than μ_*c*_ = *Nez*_*c*_*U*, with *Ne* being the Faraday constant (Avogadro constant times elementary charge) and *z*_*c*_ the charge of the particle. With this, the electrical conductivity and the diffusion constant of charged particles (e.g., in an ionic conductor) are related (σ ∝ *D*).

The general exchangeability of the variables allows the use of various measurement techniques originally developed purely for electrical applications to determine electrochemical parameters.^5,6^ The most common method is impedance spectroscopy. Here, a small periodic signal is superimposed on the applied potential *U*, and the AC response of the electric current *Ĩ*_*e*_ is analyzed.
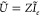
2*Z* is the complex resistivity. We highlight the use of AC by a tilde over the corresponding variable. The power of the method is due to the additional variable frequency (ω) influencing all parameters, i.e., *Z* = *f*(ω). Simplified, scanning the frequency space allows to identify the characteristic time constants of the underlying mechanisms.^5,7^ The method is a specialization of the response theory linking atomistic and macroscopic phenomena. As an established technique, most experimental cases have been treated, and solutions of *Z*(ω) to extract the corresponding parameters exist. Taking a rechargeable battery as an example, impedance spectroscopy could identify the slowest time constant to the absolute (electrochemical) charging capacitance followed by diffusion processes; the double layer capacitance and charge-transfer reactions are on medium time scales.^8^ In particular, in battery research, the different time domains are used to differentiate between the materials level, electrode level, and cell level.^9^

With this success story in mind, we apply the same principle to purely chemical reactions, concretely to heterogeneous catalysis. In heterogeneous catalysis, similar questions as have been addressed to batteries by impedance spectroscopy are as follows: What is the amount of reactants, intermediates, and products on the catalyst? How fast do they diffuse on the microscale as well as on the macroscale? And how fast do they react? As an example, we consider the methanation reaction:
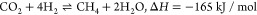
3taking place in a plug flow reactor. Various d-metals catalyze the reaction with a high degree of conversion, and the level of catalyst development is high. This makes the reaction ideal as a model system for studying the fundamental details of catalysis: the reaction mechanism of CO_2_ methanation includes adsorption and desorption as well as dissociation and association reactions.^10−13^ For Ni, Figure 1 summarizes the simplified reaction scheme as anticipated by Mohan et al.^14^

**Figure 1:**
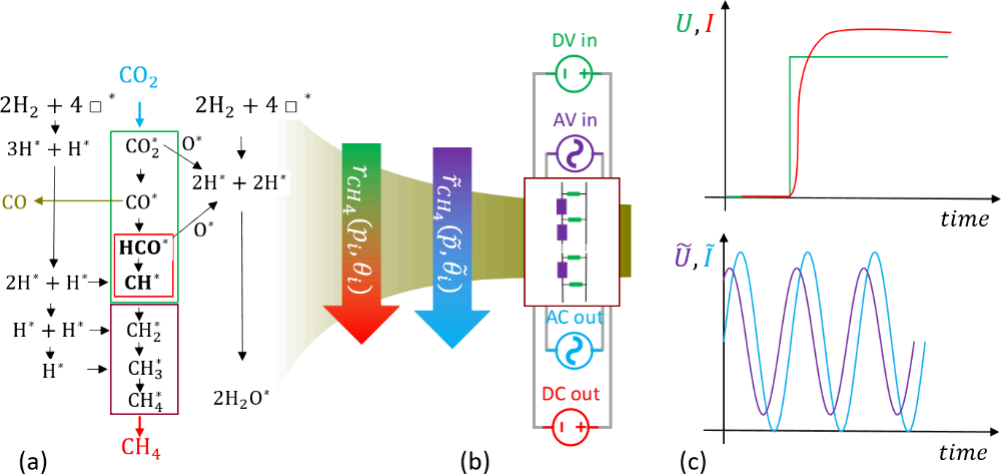
(a) Simplified reaction scheme of the CO_2_ hydrogenation to methane on Ni.^14^ The overall reaction rate to CH_4_ depends on the partial pressures *p*_*i*_ and coverage θ_*i*_ of all adsorbed species. An asterisk signifies that the molecule is adsorbed. □* represents a surface vacancy. The rate-determining reaction step is printed in bold letters. (b) Analogous electric circuits: the direct and alternating current response of a direct and alternating voltage, respectively, is the basis of impedance spectroscopy, yielding information on the underlying interaction network (transmission model). The color code links details of b and c; see text for more information. (c) Generalized approaches to time dependent phenomena by pulse measurements (top) and periodic signal perturbation (bottom).

The pathway is the basis for and from modeling, but despite advanced spectroscopy, some aspects such as the rate-determining step are still debated due to the complexity of the interaction network (see Figure 1a).^10−14^ In addition to the complexity of the chemistry taking place, a catalyst often consists of metal nanoparticles on an oxide support (here, we use Al_2_O_3_ supported Ni particles). It is thus not clear where the reaction steps take place, i.e., where the active sites are located, and where the adsorbates accumulate. This adds another degree of complexity (“materials gap”).^15^

In this Letter, we want to look at reaction mechanisms from an electrical engineer’s point of view: it can be described by an electrical transmission line model, which consists of a complex network of interacting components^16−18^ (see Figure 1b). We would like to emphasize here that, although such electric networks can be quite large, their modeling has been state-of-the-art in electric engineering for a long time.^18^ To convert an electric model into a chemical one, we rewrite the reaction rate of the formation of CH_4_, *r*_CH_4__, as Ohm’s law:
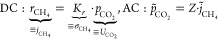
4with *K*_*r*_ being the rate constant and *p*_CO_2__ the applied CO_2_ pressure. This means that the current of generated (CH_4_) molecules *j*_CH_4__ is proportional to a potential *U*_CO_2__ with conductance σ_CH_4__. This is a strong simplification of reaction kinetics, because the pressure dependence is often nonlinear.^10−13^ The nonlinearity will be first neglected to simplify the complex analysis. However, some results bear indications of it, which may be included in future analysis.

In the steady state (≡DC), a conductance is a real number, which depends on various parameters such as temperature and hydrogen pressure and the amount of accumulated reactants, intermediates, and products (θ_*n*_). In a transmission line model, this corresponds to the amount of reciprocal complex resistivity *K*_*r*_ = |*Z*|^–1^ (see SI, section 5, for a tutorial explanation of the concept).^5^

It is worth noting that, although we know that σ_CH_4__ depends on the (time-dependent) coverage by intermediates θ_*i*_, this dependence does not enter eq 4 directly, and thus cannot be studied by steady state methods. In electrical engineering, the same is true for the application of DC currents on electric circuits containing capacitive or inductive elements. However, the “hidden” components can be measured by time-dependent methods. The simplest is the application of a step function of the potential resulting in an exponential response of the current (Figure 1c), e.g., to measure the capacitance of a capacitor. The catalysis analogue of the capacitance of an electrical capacitor is the number of molecules adsorbed on a catalyst. Indeed, pulse measurements are used to derive this parameter. An elegant alternative is to apply an alternating potential (“AC”). This is the basis of impedance spectroscopy, which we apply here on catalysis.

The application of periodic signals in catalysis is technically straightforward. Mass flow controllers control gas flows of the order of 100 cm^3^ min^–1^ on a subsecond time scale. More demanding is the detection of the products. In this paper, we use a Fourier transform infrared (FTIR) spectrometer (Bruker, Germany) measuring the infrared absorption of product gases in an IR-gas cell (see Supporting Information Figure S1). The cell plus pipework has a volume of ≤50 cm^3^. We thus run the setup at a total flow of >200 cm^3^ min^–1^, which limits the time resolution to around Δτ ≃ 20 s. The time τ = 20 s is sufficient for one full scan by the FTIR spectrometer and is thus taken as the basic time constant of the experimental setup.

The catalytic reaction takes place in a stainless steel tube reactor at various temperatures. The catalyst used was Al_2_O_3_-supported Ni (see Supporting Information). A space velocity of 44 000 h^–1^ using correspondingly small catalyst volumes was used. The value is high enough to not add a substantial time delay and limits the total conversion (see results later). Experiments were run at an overstoichiometric H_2_/CO_2_ ratio of 6:1. We did not modulate the H_2_ flux to avoid understoichiometry. We modulated the CO_2_ partial pressure between 0 and 100% of the 6:1 ratio by using a *rectangular* periodic pattern. The rectangular pattern is first a technical necessity: the controlling of a sinusoidal flow pattern is technically challenging. However, it appeared that the deconvolution of the resulting product signal into the fundamental frequency and overtones yields additional information (Supporting Information, Figure S3).

Figure 2a,b show the response of the system to rectangular periodic system at two different frequencies *f*. As we are mainly interested in the time lag (phase shift Φ = 2π(*t*_CH_4__ – *t*_CO_2__)*f*) and amplitude ratio *r* = *r*_CO_2__/*r*_CH_4__, fitting the signal with a sine function is sufficient:
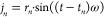
5The time lags *t*_*n*_ are related to the time constant derived from exponential fit to the response from a nonperiodic pulse (see Figure 2a and SI, section 5). We do not elaborate on these results further. However, the time constant of the switch-off response is a first indication of the applicability of CIS experiments. Such switch-off behavior can be measurable with many catalytic setups. If the time constant is much smaller than the smallest periodic time constants of the CIS setup, then CIS experiments will not make sense. Such a measurement can thus be seen as a feasibility study to be performed on an unknown reaction before costly CIS experiments.

**Figure 2:**
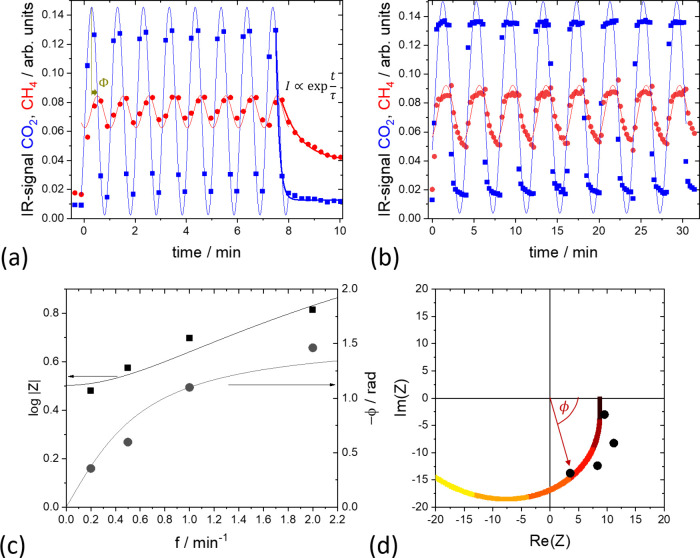
(a and b) Modulated CO_2_ signals and CH_4_ response at two different frequencies. Squares and dots are experimental data; lines are fits to eq 5 (except the exponential decay at the end of an experiment). (c) Bode plot of the frequency dependence of the amplitude |*Z*| = *r* and phase ϕ extracted from multiple measurements such as shown in a and b; here, for *T* = 250 °C. (d) Nyquist plot of the data in c shown as full circles; the colored line is the model fit (eq 2). Yellow indicates high frequencies, and black indicates low frequencies.

The aim of this paper is to demonstrate the concept of “catalytic impedance spectroscopy” to gain insights into reaction mechanisms of catalytic reactions. Figure 1a gives an overview of the various intermediate steps to be expected for the CO_2_ reduction to methane. We simplify this reaction into two steps: the adsorption and dissociation of CO_2_ up to the rate-determining step and further reaction into CH_*x*_ formation and desorption of CH_4_. We also consider desorption of CO. To model it with three steps, the rate constant *k* summarizes all incoming terms, *k*_d_ the desorption of intermediates, and *k*_*c*_ all steps leading to desorption of CH_4_. We are aware that several intermediates are present of the surface but use θ_*n*_ as the variable for the rate-determining intermediate. The change of its coverage is then
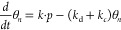
6simplifying that adsorption and subsequent dissociation of CO_2_ is proportional to the applied pressure *p*. The CH_4_ particle current is *j* = −*k*_*c*_θ_*n*_. Inserting this and rearranging the equation yields the differential equation
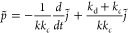
7The steady state eq 4 is changed to the AC case, and with *j̃* ∝ exp *i*ω*t*, we obtain the “AC resistance” (impedance) *Z* = *p̃*/*j̃* analogous to the electric relation eq 2:
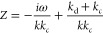
8This is equivalent to an inductive electric circuit (RL-circuit) with a negative phase angle ϕ
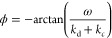
9The negative phase angle is evident directly from the raw measurements shown in Figure 2a and b: the CH_4_ response is ahead of the CO_2_ stimulation. For simplicity (and following technical convention), we discuss −ϕ. The amplitude *r* is
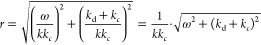
10Both amplitude and phase angle depend on frequency. In impedance spectroscopy, this behavior is usually shown in the so-called Bode diagram (Figure 2c). Simplified, the phase increases with frequency and approaches zero at zero frequency, i.e., steady-state conditions. To highlight the phase dependence, the complex impedance is plotted in a Nyquist plot exhibiting the circle (Figure 2d), which is expected for an inductive electric circuit (see also SI, section 5).^5^

The discussion above aimed at setting the fundamentals of modeling a catalytic reaction within the mathematical framework of impedance spectroscopy. In the following, we discuss how the parameters derived from the empirical model contribute to an understanding of the process. The parameters *k*, *k*_d_, and *k*_*c*_ are empirical, but conclusions can be drawn from the temperature dependence. Thus, CIS measurements were performed at various temperatures. Figure 3 plots the amplitude and phase of the complex resistance at various temperatures. The phase differences declined with higher temperatures. Similarly, the changes of the amplitude on frequency weakens. The absolute value, however, decreases markedly. The relationships between the parameters can be seen by comparing *kk*_*c*_ and *k*_d_ + *k*_*c*_ as a function of temperature derived by fitting to eqs 9 and 10. The parameter sets *k*_d_ + *k*_*c*_ (black squares and red dots in Figure 5) are the same regardless of whether they are derived from the amplitude or from the phase, showing the robustness of the model. The parameter product *kk*_*c*_ scales perfectly with the space–time CH_4_ yield measured independently on the same sample (Figure 5). This observation supports the reasonable assumption that the amplitude is *r* ∝ (*kk*_*c*_)^−1^ (eq 10) and that the results of CIS can be compared to the (steady-state) rate constant *R* ∝ |*K*_*r*_|. This corresponds to a negligible desorption of CO (*k*_d_ ≪ *k*_*c*_), which is indeed observed.

**Figure 3:**
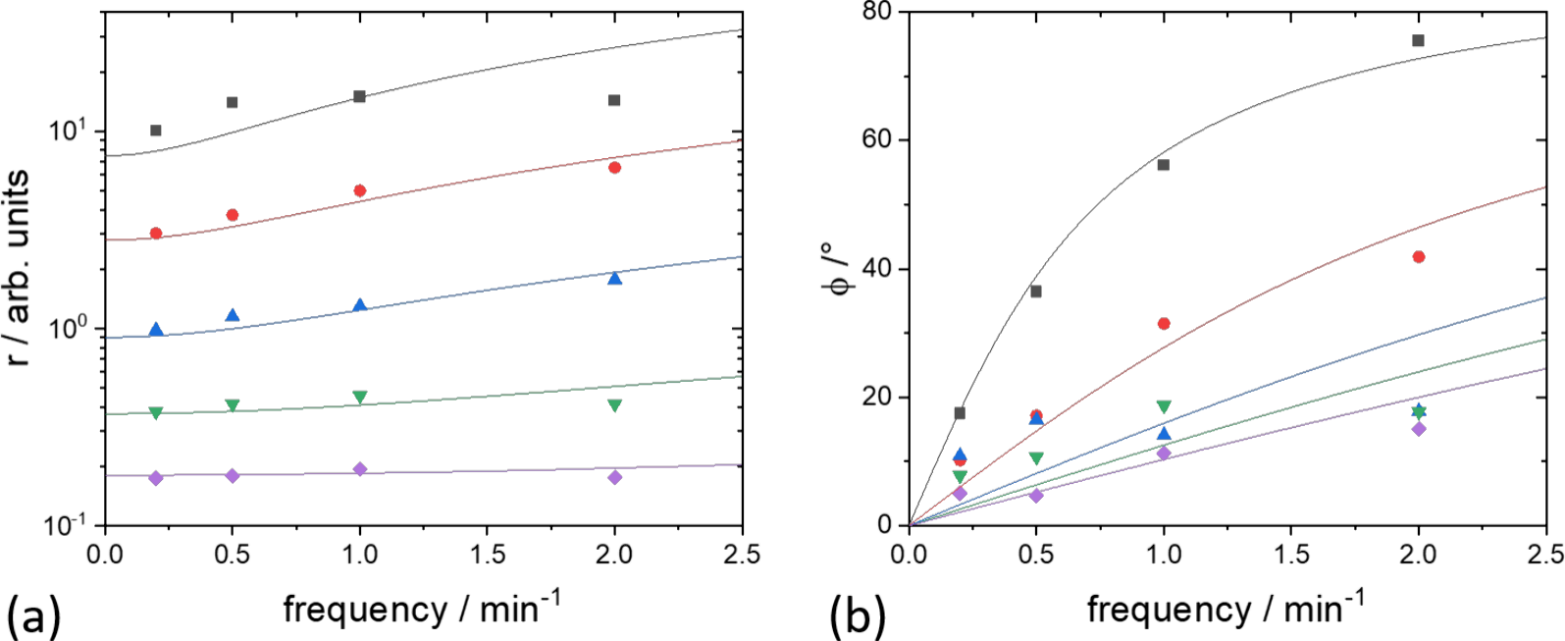
Frequency dependence of the amplitude (a) and phase (b) for temperatures *T* = 209°, 236°, 261°, 286°, and 310 °C from top (black) to bottom (violet), respectively. Squares and dots are experimental data; lines are fits to eqs 10 and 9.

The small amount of CO impedes its use for impedance spectroscopy due to the bad signal-to-noise ratio. At low temperatures, CO is not detected. At higher temperatures, the degree of CO desorption increases. Figure 4 shows measurements at *T* = 350 °C for CO_2_, CH_4_, and CO. Within noise, the CO signal follows that of CO_2_, but it is significantly different from the CH_4_ signal. With *r*_CO_ = *k*_d_θ_CO_, we can conclude that the rate-determining step cannot be CO to subsequent intermediates: in this case, *r*_CO_ would follow the CH_4_ signal, which is not the case.

**Figure 4:**
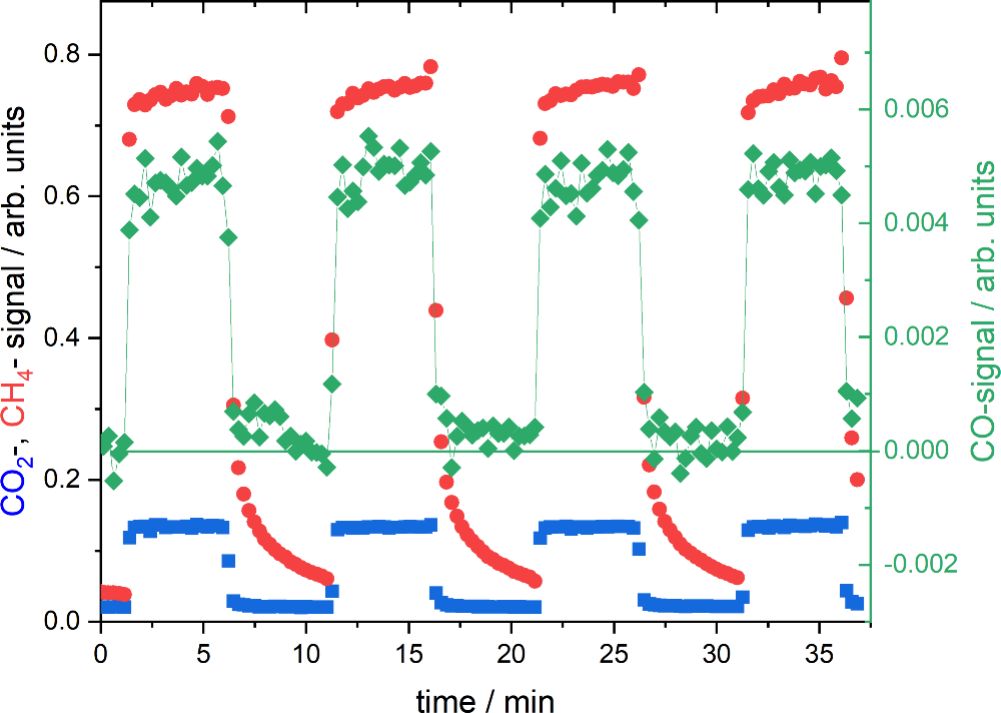
Modulated CO_2_, CH_4_, and CO signals at *T* = 310 °C.

Further insights must come from modeling, e.g., based on DFT calculations.^14^ The theoretical results can be simplified as follows: CO_2_ → CO_2_^*^ → CO* → CHO* → CH* → CH_2_^*^ → CH_3_^*^ → CH_4_, with the rate-determining step being CHO* → CH*. This is in perfect agreement with the results of our CIS measurements (Figure 5). There is a further interesting detail from modeling: CO* is calculated to be one of the most abundant intermediates, although its further reaction is not the rate-determining step. The relatively large coverage is due to its strong chemisorption, which can even cause so-called CO poisoning.^19^ The CIS measurements indicate that CO formation is linked to the adsorption process, and its formation is not rate-determining for CH_4_ production. This surprising result demonstrates the advantage of a dynamic rate analysis such as CIS over catalyst characterization focusing on detecting adsorbates.^20^

**Figure 5:**
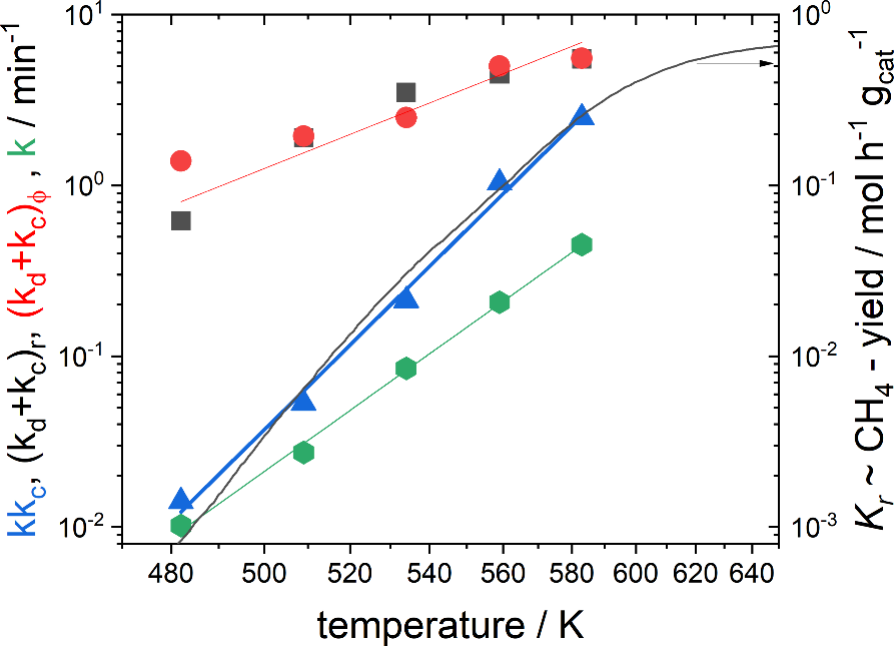
Arhenius plot of rate constants extracted from CIS measurements compared to temperature dependence of the CH_4_ yield (∝ *K*_*r*_) in steady-state.

An Arrhenius analysis yields the apparent activation energies of the individual processes (see Figure 5 and Supporting Information, Figure S2). *E*_*AA*_ of the overall process is 130 ± 10 kJ/mol, which is in agreement with data on similar Al_2_O_3_ supported Ni catalysts^21^ (see also discussion on activation energy in the Supporting Information). With *R* ∝ *kk*_*c*_, it can be deconvoluted into *E*_*AA*_ = *E*_*k*_ + *E*_*k*_*c*__ = 95 + 35 kJ/mol. The activation energy *E*_*k*_*c*__ can be derived from the amplitude as well as phase dependence (Figure 5, black squares and red spheres). The values agree very well within noise, confirming the robustness of the analysis method.

An activation energy of *E*_*k*_ ≫ *E*_*k*_*c*__ means that the adsorption/dissociation of CO_2_ is the dominant process in the overall methanation reaction. We avoid calling this the “rate-limiting” step because then the activation energy would be *E*_*AA*_ ≡ *E*_*k*_ and *K*_*r*_ ∝ *k*, which is not the case. The underlying step determines the optimum path but does not limit the overall rate; hence, it is the “rate-determining step”. However, the value *E*_*k*_ = 95 kJ/mol coincides with the free energy barriers of 104 and 88 kJ/mol for the formation of CH* and CH_2_^*^ via HCO* and CH_2_O* dissociation, respectively, which were identified as potential rate-determining steps.^14^

The existence of a rate-determining step explains the observed “two-step” behavior as modeled by eq 6. The model assumes that there is one intermediate that determines the formation of CH_4_ (described by θ_*n*_), which is the intermediate produced by the rate-determining step. By comparison with theory,^14^ we can assign it to HC*. The reaction steps before this step are not rate-determining but contribute to the “first step” *k*. The steps after it add up to *k*_*c*_. A better time resolution may make further deconvolution of these two steps possible.

The breakdown into two main reaction steps is experimentally corroborated by the fact that the reaction rate is enhanced by the removal of water (sorption enhanced catalysis^22^). Apart from a general coverage effect, water removal can only affect the elementary steps CO_2_^*^ + 2H* → CO* + H_2_O and HCO* + 2H* → CH* + H_2_O (see also Figure 1a). The latter is the rate-determining step according to calculations.^14^ Further experimental indication of HCO* → CH* comes from the fact that C-deposition generated by direct disscociation of CO to C* is usually not observed under methanation conditions.^23^

In conclusion, this paper introduces the concept of catalytic impedance spectroscopy to study catalytic reactions, in particular to deconvolute the reaction mechanism. It may be seen as a further development of modulation excitation spectroscopy (MES). In contrast to MES, weight is given to the time dependence of the process as derived from a complex number analysis, and less to the characterization of intermediates on the surface by advanced spectroscopy.^2^ In fact, the challenge lies in detecting the products as fast as possible with a high signal-to-noise ratio, enabling the complex number analysis. Simple sensors, such as thermal conductivity detectors (TCD), are not able to unambiguously identify the chemical compounds involved. However, with their fast response,^24^ the dynamic range of CIS can be extended markedly to the high frequency range, if combined with a chemically selective detector (such as the FTIR spectrometer as used here) characterizing the chemistry at low frequencies and thus the corresponding signal change of the TCD. We envision that a simplified CIS analysis can also be used to characterize large scale reactors. The concept must then consider the variable space in addition to time, which is beyond the scope of this paper.

The idea of CIS was inspired by the concept of resonance catalysis by Dauenhauer et al.^25^ Here, the reaction yield is increased beyond the limit given by the scaling laws when the reaction is run at a specific resonance frequency. However, the optimum modulation frequency is much higher than that achieved in typical catalysis systems such as the present one. Our approach explains why: the AC response of the system is “dampened” by the accumulation of the adsorbates. Nevertheless, the CIS method may help to develop catalysts or processes with an appropriately fast response.

Despite its insights, this study is not without limitations, paving the way for future explorations. A wider frequency as utilized in this pilot study will give more details. Concretely for the methanation reaction, we may distinguish adsorption and dissociation of CO_2_ into the various intermediates and their further reaction if utilized at faster frequencies. This is particularly evident in the Nyquist plot (Figure 2d), where the experimental data cover only a small part of the theoretically expected behavior. The use of modulated labeling (modulated H_2_/D_2_ exchange) may give insights into the hydrogenation of CH* to CH_4_. Better resolved impedance spectra will need more sophisticated models than those used here. We foresee a user-friendly software approach analogous to electrochemical impedance spectroscopy packages, in which measurement, simulation, and fitting are combined.

The methanation reaction was chosen as a well-known model reaction. There are a number of reactions, in which the product selectivity depends on the residence time of the intermediates in the reactor, such as the methanol-to-olefin reaction.^26^ Apart from an analysis of the reaction mechanism by CIS, modulation of the input may be used to actively control the selectivity of the reaction.

## Supporting Information Available

Experimental procedures and additional information on FTIR gas analysis, CO_2_ conversion and calculation of activation energy, and Fourier transformation of signals (PDF)
Transparent Peer Review report available (PDF)
