# Supplementary material for: Catalytic Impedance Spectroscopy: Concept and Application on CO2 Methanation
Source: J Phys Chem Lett. 2024 Oct 11;15(42):10451–6. doi: 10.1021/acs.jpclett.4c02442 (PMC11514020; doi:10.1021/acs.jpclett.4c02442)
Supplement: Supplementary file 1 — jz4c02442_si_001.pdf [file jz4c02442_si_001.pdf]

Supporting Information: Catalytic Impedance spectroscopy: concept and application on CO_2 methanation

Andreas Borgschulte,[*,†,‡] Marco Achermann,[¶] and Marin Nikolic[†,§]

†*Empa - Swiss Federal Laboratories for Materials Science & Technology, Laboratory Chemical Energy Carriers and Vehicle Systems Laboratory, Überlandstrasse 129, CH 8600 Dübendorf Switzerland*

‡*Department of Chemistry, Zurich University, Winterthurerstrasse 190, CH 8057 Zürich*

¶*Kantonsschule Reussbühl Luzern, Ruopigenstrasse 40, CH 6015 Luzern, Switzerland*

§*Department of Chemistry, Zurich University, Zurich*

E-mail: andreas.borgschulte@empa.ch

Phone: +41 58 765 4639

1. Catalyst preparation

Aluminum oxide support pellets (43832, *Thermoscientific*) were calcinated at 500 ℃ for 5 hours (heating rate 5 ℃ min^{-1}) before wet impregnation. $Ni(NO_3)_2 \cdot 6H_2O$ (*Sigma Aldrich*) at a loading of 10 %-wt (Ni) was added to the precalcinated Al_2O_3 pellets, dissolved in deionized water and stirred at 60 ℃ for 24 hours. The calcination of the impregnated samples was done in air with an oven temperature set to 450 ℃ for 5 hours (heating rate 5 ℃ min^{-1}). The nickel loading, as determined by weighing the sample before and after impregnation, was 4.3 %-wt.

2. Gas analysis by FTIR

The exhaust gas containing the CO_2, CH_4 and traces of CO was analyzed by FTIR equipped with a 7 cm gas cell kept at room temperature. The peak heights of the indicated peaks (Fig. S1) were used as a measure of concentration. For conversion and absolute CH_4 measurements (Fig. S2 and Fig. 4 in the main document), the signals were converted into concentration with gas calibration.

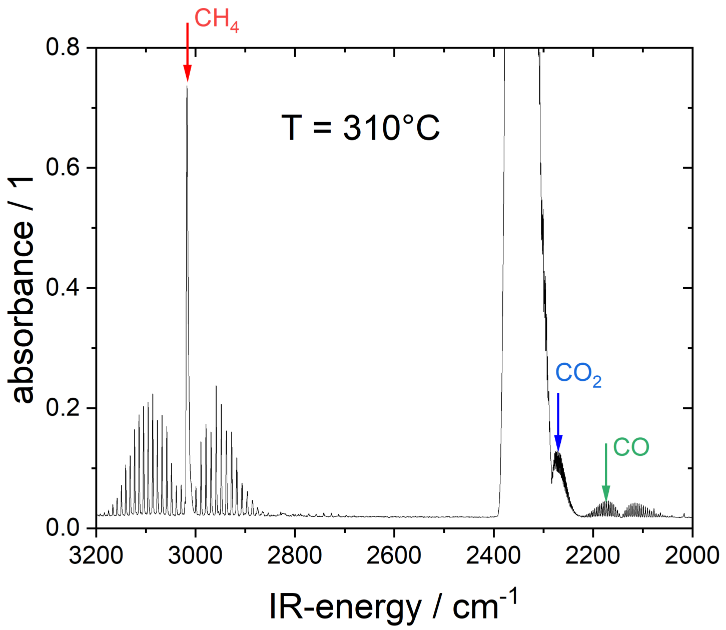

Figure S1: IR-signal at highest CO production ($T = 310°C$).

In principle, the gas IR contains information on the water formed during methanation. However, water adsorbs/condenses at the walls of the analysis system blurring the time resolution of the analysis. It is thus removed from the analysis stream directly after the reactor using a cooling trap.

3. Conversion and activation energy

Figure S2 compares the CO_2 conversion over temperature at $p_{total} = 1$ bar, H_2:CO_2 = 6:1, and a space velocity of 44000 h^{-1} with literature data. In principle, an apparent activation

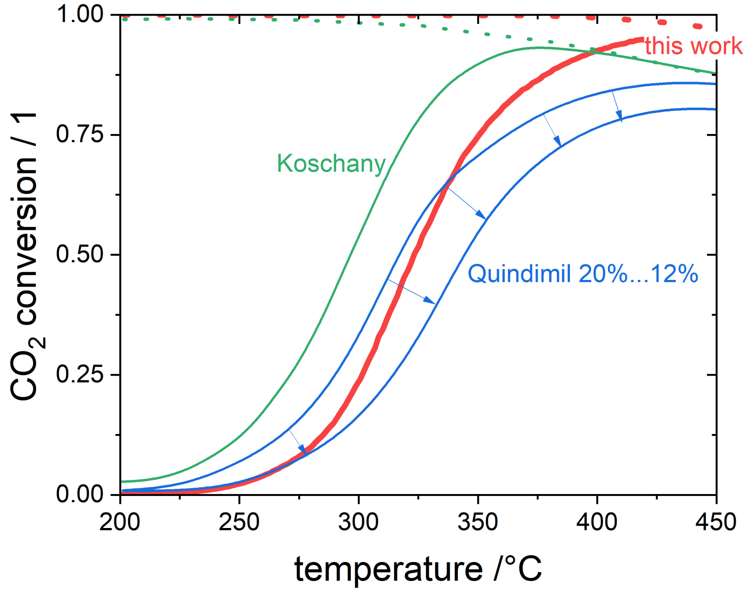

Figure S2: CO_2 conversion over temperature. Full lines are measured values, dashed lines correspond to the maximum yield limited by thermodynamics (from Ref.[S1]) at the corresponding reaction conditions. Koschany is taken from Ref.,[S2] Quindimil from Ref.[S3] Quindimil et al. have have studied Ni@Al_2O_3 with various Ni contents as indicated by the arrows.

energy can be derived from this data by

$$E_{AA} = -R_A \frac{\partial \ln R_{CO_2}}{\partial(1/T)} \tag{1}$$

With this, we obtain E_{AA,CO_2} =126 kJ/mol, which is in good agreement with Quindmill et al.[S3] on similar catalysts. However, several other publications give other values.[S2] Figure S2 indicates that this is in part due the different conditions used (pressure, flow, stoichiometry). Various studies (e.g. Herwijnen et al.[S4]) redefine the activation energy to make to release the dependence on thermodynamic conditions. Unfortunately, as the models used differ from publication to publication,[S2,S4] the model activation energies are not comparable. Further-

more, the macro and micro structure of the catalyst influence the result. Concretely, for the study we have used 2 mm beads, which show small differences to the same but ground catalyst.

4. Fourier transformation of rectangular modulated signals

Figure S3 shows the Fourier transform of the rectangular CO_2 and CH_4 signal. In addition

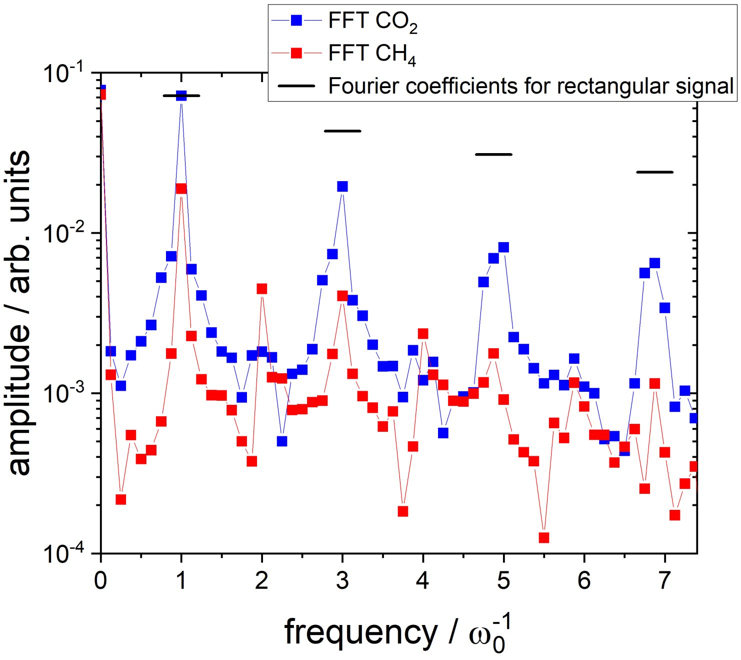

Figure S3: Fourier transform of the of rectangular modulated signals. The Fourier transform of a perfect rectangular signal should consist only of odd overtones with coefficients shown by bars (normalized to the one of the ground frequency).

to the expected odd overtones, the CH_4 signal contains even overtones indication of a highly non-linear behavior.

5. Analogy between electric circuits and adsorption phenomena

Despite being based on completely different interacting species, i.e., electrons and molecules in electric circuits and adsorption, respectively, the underlying math is practically identical. We discuss here the case of storage of electrons in capacitors, and storage of molecules on surfaces.

The capacitance C of a capacitor is defined as charge Q per voltage U

$$C = \frac{Q}{U} \rightarrow C = \frac{dQ}{dU}, \tag{2}$$

best described as a differential to take into account a possible non-linearity of the process.[S5]

In the simplest case, adsorption is described by Henry's law

$$\theta = K_H p \rightarrow K_H = \frac{\theta}{p} \rightarrow K_H = \frac{d\theta}{dp}. \tag{3}$$

Again, the differential form considers the non-linearity of adsorption phenomena, which are more common in adsorption than in capacitors (Langmuir, BET,...). K_H is the adsorption constant, which in analogy to the capacitor could also be called adsorption capacitance.

A straightforward method to approach the adsorption capacitance is applying a pressure on a pressure cylinder resulting in a temporal response of the system, i.e., the coverage changes by

$$\frac{d\theta}{dt} = kp \tag{4}$$

$$\frac{d\theta}{dt} = \frac{d\theta}{dp}\frac{dp}{dt} = K_H \frac{dp}{dt} = kp \tag{5}$$

This differential equation is easily solved

$$\int_{p_0}^{p(t)} \frac{dp}{p} = \int_0^t \frac{k}{K_H} dt \rightarrow \frac{p(t)}{p_0} = e^{-\frac{k}{K_H}t} \tag{6}$$

The equation is the expected exponential function.

In an electric RC circuit, the analog description is

$$\frac{dQ}{dt} = R^{-1}U \rightarrow \frac{dQ}{dU}\frac{dU}{dt} = C\frac{dQ}{dt} = R^{-1}U, \tag{7}$$

where R is the resistivity and thus

$$\frac{U(t)}{U_0} = e^{-\frac{1}{RC}t}, \tag{8}$$

yielding the expected decay curve of a (dis-)charging a capacitor.

The analogy also explains the parameter k, which we did not define in eq. 4. It is a kind of conductivity (inverted resistivity), corresponding to the "rate constant" of the adsorption process described by the very simple chemical reaction

$$A \rightarrow A^* \tag{9}$$

The model used in the main body of the paper (eq. (4), and more specifically eq. (6)) also considers conversion of adsorbed species on the surface, which is of course not possible in electric circuits. We anyway can describe it mathematically by an "effective" conductivity.

The next step is to unify the steady-state (= DC) conductivities with the time dependent parameters. There is an elegant solution for periodic excitations from electric engineering. This is the concept of complex resistivity $Z^{-1} \rightarrow K_r$, where a resistivity is a complex number described by either an amplitude r and phase ϕ or by a real x and imaginary number iy (so-called impedance):[S5,S6]

$$Z = re^{i\phi} = x + iy;\ r = \sqrt{x^2 + y^2},\ \phi = \arg(Z) \tag{10}$$

In case of the RC-circuit, the complex resistivity becomes

$$Z = R + i\frac{1}{\omega C} \tag{11}$$

The outgoing signal is then "weakened" by the factor described the amplitude r, and phase between in- and outgoing signal shifted by ϕ:

$$\tilde{I} = \tilde{U}\frac{1}{Z} \tag{12}$$

On a first view, this looks like a complication, as the corresponding differential equation can be solved directly. However, for more complex networks ("transmission lines"), the complex resistivities can be added just like you do with "normal" resistivities employing Kirchhoff's circuit laws.[S5]

For the simple adsorption case, the complex resistivity becomes

$$Z_{ad} = \frac{1}{k} + i\frac{1}{\omega K_H}, \tag{13}$$

which translates the periodic input pressure $\tilde{p}$ into the output flow $\tilde{j}$:

$$\tilde{j} = \tilde{p}\frac{1}{Z_{ad}} \tag{14}$$

This is the mathematical operation manual to extract relevant materials parameters from periodic excitation experiments.

Interestingly, the model for the methanation reaction (eq. (6)) does not include a complex resistivity of the "capacitor" kind, but one which has the complex behavior analog to an inductive element:[S6]

$$Z_{el:inductivity} = i\omega L \quad Z_{chem} = -i\omega k_1 + k_2 \tag{15}$$

The constants k_1 and k_2 are defined in more detail in the main text. The qualitative expla-

nation is that the intermediate accumulates similar to the built up of a magnetic field.

Finally, various other impedance models have been developed for electrochemical systems. In particular diffusion as a relevant reaction step also in catalysis can be modelled, e.g., by the Warburg diffusion model.[S7]

References

(S1) Gao, J.; Wang, Y.; Ping, Y.; Hu, D.; Xu, G.; Gu, F.; Su, F. A thermodynamic analysis of methanation reactions of carbon oxides for the production of synthetic natural gas. *RSC Adv.* **2012**, *2*, 2358–2368.

(S2) Koschany, F.; Schlereth, D.; Hinrichsen, O. On the kinetics of the methanation of carbon dioxide on coprecipitated NiAl(O). *Applied Catalysis B: Environmental* **2016**, *181*, 504–516.

(S3) Quindimil, A.; De-La-Torre, U.; Pereda-Ayo, B.; Davó-Quiñonero, A.; Bailón-García, E.; Lozano-Castelló, D.; González-Marcos, J. A.; Bueno-López, A.; González-Velasco, J. R. Effect of metal loading on the CO2 methanation: A comparison between alumina supported Ni and Ru catalysts. *Catalysis Today* **2020**, *356*, 419–432.

(S4) van Herwijnen, T.; van Doesburg, H.; de Jong, W. A. Kinetics of the methanation of CO and CO_2 on a nickel catalyst. *J. Catal.* **1973**, *28*, 391–402.

(S5) Hambley, A. R. *Electrical Engineering: Principles and Applications*; Pearson Education, 2011.

(S6) Lazanas, A. C.; Prodromidis, M. I. Electrochemical Impedance Spectroscopy - A Tutorial. *ACS Measurement Science Au* **2023**, *3*, 162–193.

(S7) Huang, J. Diffusion impedance of electroactive materials, electrolytic solutions and

porous electrodes: Warburg impedance and beyond. *Electrochimica Acta* **2018**, *281*, 170–188.
