# Supplementary material for: Catalytic Impedance Spectroscopy: Concept and Application on CO2 Methanation
Source: J Phys Chem Lett. 2024 Oct 11;15(42):10451–6. doi: 10.1021/acs.jpclett.4c02442 (PMC11514020; doi:10.1021/acs.jpclett.4c02442)
Supplement: Supplementary file 2 — jz4c02442_si_002.pdf [file jz4c02442_si_002.pdf]

jz-2024-02442d.R1

Name: Peer Review Information for "Catalytic Impedance Spectroscopy: Concept and Application on CO₂ Methanation"

First/Second/Third Round of Reviewer Comments

Reviewer: 1

Comments to the Author

The present manuscript introduces the concept of Catalytic Impedance Spectroscopy (CIS) to extract valuable mechanistic information from catalytic reactions. The concept is applied to CO2 methanation to validate the theoretical foundations. Certainly, it is a very interesting concept, with important experimental challenges, but provides a novel methodology for the community studying reaction mechanisms in catalytic reactions. However, there are some issues, which need to be addressed before the manuscript can be accepted for publication. More detailed comments are provided below:

1) Page 5, in the simplified reaction scheme in Figure 1(a) the symbol “4*” is not understood.
2) Page 5, the authors list a couple of references (16, 17) on the transmission line model. Some additional references could be added, for example J. Phys. Chem. B 2002, 106, 2, 325–333.
3) In page 6 the authors claim: “In steady state (≡DC), a conductance is a real number, which depends on various parameters such as temperature and hydrogen pressure, and the amount of accumulated reactants, intermediates and products (θ_n). In a transmission line model, this corresponds to the reciprocal complex resistivity Kr = $|Z|^{-1}$.” However, this needs further explanation for the non-expert readers.
4) Additionally, in page 6, the authors claim: “The catalysis analogue of the capacitance of an electrical capacitor is the amount of molecules adsorbed on a catalyst.” But it would be more appropriate referring to the capacitance as a differential quantity, dQ/dV, which would translate into a differential quantity in the chemical analogue, dθ/dP?
5) Page 8, the authors claim: “...the time constant of the switch-off response is a first indication of the applicability of the experiments to be performed on an unknown reaction before starting time costly CIS experiments”. This sentence needs further clarification for the non-expert readers.
6) In page 8, Figure 2 caption needs to include (d) for the Nyquist plot.
7) In Eq 7, the authors use a notation which has not been introduced in the main text (a dot over the quantity), this should be clearly explained, at least in the SI. The authors should also revise Eq 7.

8) In page 10, the authors claim: “To highlight the phase dependence, the complex impedance is plotted in a Nyquist plot exhibiting the circle (Fig. 2 (c)), which is expected for an inductive electric circuit.5” The authors mean Figure 2(d).
9) Furthermore, the experimental Nyquist plot (symbols) does not follow the solid line. What is the meaning of this solid line?
10) Page 11. The authors claim: “The parameter product kkc scales perfectly with the space-time CH_4 yield measured independently on the very same sample (Fig. 5). This observation supports the reasonable assumption that the amplitude is $r \propto (kkc)^{-1}$ (eq. 10) and the (steady-state) rate constant is $R \propto Kr$”. Could the authors elaborate more on this claim to clarify the meaning and implications? It is not clear for this reviewer.
11) In the conclusions, the authors claim: “In fact, the challenge lies in detecting the products as fast as possible with a high signal to noise ratio enabling the complex number analysis”. In this context, the authors could further elaborate on the practical application of the concept and on the main existing barriers and how to overcome them.

Author's Response to Peer Review Comments:

We thank the reviewer for the careful reviewing and the many suggestions. We have the feeling that the re-viewer shares our enthusiasm on the concept, and her/his suggestions helped to improve the readability. However, some suggestions, which we do appreciate very much, would expand the "letter" very much. We mainly extended the SI to provide this additional information. Important changes are highlighted in red in the manuscript.

R: The present manuscript introduces the concept of Catalytic Impedance Spectroscopy (CIS) to extract valuable mechanistic information from catalytic reactions. The concept is applied to CO_2 methanation to validate the theoretical foundations. Certainly, it is a very interesting concept, with important experimental challenges, but provides a novel methodology for the community studying reaction mechanisms in catalytic reactions.

However, there are some issues, which need to be addressed before the manuscript can be accepted for publication. More detailed comments are provided below:

1) Page 5, in the simplified reaction scheme in Figure 1(a) the symbol “4*” is not understood.

A: We replaced it with a square^* and added its description in the caption.

2) Page 5, the authors list a couple of references (16, 17) on the transmission line model. Some additional references could be added, for example J. Phys. Chem. B 2002, 106, 2, 325–333.

A: Implemented. See also SI, section 5.

3) In page 6 the authors claim: “In steady state (≡DC), a conductance is a real number, which depends on various parameters such as temperature and hydrogen pressure, and the amount of accumulated reactants, intermediates and products (θ_n). In a transmission line model, this corresponds to the reciprocal complex

resistivity Kr = |Z|−1.” However, this needs further explanation for the non-expert readers.

A: First, there is typo, it is Kr= Z^-1. Second, thank you for the suggestion; in fact, the knowledge behind this seemingly simple relationship is vast and not always so simple. However, adding this here is too much for a "letter"; we thus refer to the SI, where added a full chapter on it. (section 5, for tutorial explanation of the concept).

4) Additionally, in page 6, the authors claim: “The catalysis analogue of the capacitance of an electrical capacitor is the amount of molecules adsorbed on a catalyst.” But it would be more appropriate referring to the capacitance as a differential quantity, dQ/dV, which would translate into a differential quantity in the chemical analogue, dθ/dP?

A: This is a very nice suggestion. However, as it expands the manuscript, we added a full section in the SI, in which this idea is elaborated (section 5). It actually matches nicely the tutorial explanation of impedance in catalysis as suggested by question 3.

5) Page 8, the authors claim: “...the time constant of the switch-off response is a first indication of the applicability of the experiments to be performed on an unknown reaction before starting time costly CIS experiments”. This sentence needs further clarification for the non-expert readers.

A: Yes, this part is a little short, and is a result of the history behind the idea: we always wondered why it took so long before the system was properly "purged" after an experiment. Now we can make use of the effect. We changed/added: "However, the time constant of the switch-off response is a first indication of the applicability of CIS experiments. Such a switch-off behavior is measurable with many catalytic setups. If the time constant is much smaller than the smallest periodic time constants of the CIS setup, CIS experiments will not make sense. Such a measurement can thus be seen as a feasibility study to be performed on an unknown reaction before starting time costly CIS experiments."

6) In page 8, Figure 2 caption needs to include (d) for the Nyquist plot.

A: Added, together with a description of the line (question 9).

7) In Eq 7, the authors use a notation which has not been introduced in the main text (a dot over the quantity), this should be clearly explained, at least in the SI. The authors should also revise Eq 7.

A: We changed the dot f (Newton's notation) to the Leibniz' notation df/dx, which is more common.

8) In page 10, the authors claim: “To highlight the phase dependence, the complex impedance is plotted in a Nyquist plot exhibiting the circle (Fig. 2 (c)), which is expected for an inductive electric circuit.5” The authors mean Figure 2(d).

A: Yes, thank you.

9) Furthermore, the experimental Nyquist plot (symbols) does not follow the solid line. What is the meaning of this solid line?

A: We added in the caption: "d) Nyquist plot of the data in (c) shown as full circles; the colored line is the model fit (eq. 2). Yellow indicates high frequencies, black low frequencies."

And in the main text: " This is particularly evident in the Nyquist plot (Fig. 2 (d)), where the experimental data cover only a small part of the theoretically expected behavior." We already mentioned in the original manuscript (conclusion) that the model should be improved.

10) Page 11. The authors claim: “The parameter product kkc scales perfectly with the space-time CH_4 yield measured independently on the very same sample (Fig. 5). This observation supports the reasonable assumption that the amplitude is $r \propto (kkc)^{-1}$ (eq. 10) and the (steady-state) rate constant is $R \propto Kr$”. Could the authors elaborate more on this claim to clarify the meaning and implications? It is not clear for this reviewer.

A: With this sentence we just wanted to make clear that CIS does not measure any "nonsense". We re-phrased: "This observation supports the reasonable assumption that the amplitude is $r \propto (kkc)^{-1}$ (eq. 10) and that the results of CIS can be compared to the (steady-state) rate constant $R \propto |Kr|$."

The real outcome of CIS is discussed later (that you can "deconvolute" the rate constant).

11) In the conclusions, the authors claim: “In fact, the challenge lies in detecting the products as fast as possible with a high signal to noise ratio enabling the complex number analysis”. In this context, the authors could further elaborate on the practical application of the concept and on the main existing barriers and how to overcome them.

A: With pleasure: "Simple sensors such as thermal conductivity detectors (TCD) are not able to unambiguously identify the chemical compounds involved. However, with their fast response,24 the dynamic range of CIS can be extended markedly to the high frequency range, if combined with a chemically selective detector (such as the FTIR spectrometer as used here) characterizing the chemistry at low frequencies and thus the corresponding signal change of the TCD. We envision that a simplified CIS analysis can also be used to characterize large scale reactors. The concept must then consider the variable space in addition to time, which is beyond the scope of this paper. "
